# An overview of menopausal oestrogen–progestin hormone therapy and breast cancer risk

**DOI:** 10.1038/sj.bjc.6602617

**Published:** 2005-05-17

**Authors:** S A Lee, R K Ross, M C Pike

**Affiliations:** 1 Department of Preventive Medicine, Norris Comprehensive Cancer Center, University of Southern California Keck School of Medicine, 1441 Eastlake Avenue, Topping Tower 4423, Los Angeles, CA 90033-0800, USA

**Keywords:** meta-analysis, hormone therapy, breast cancer

## Abstract

Results from the Women's Health Initiative (WHI) trial support findings from observational studies that oestrogen–progestin therapy (EPT) use is associated with an increase in breast cancer risk. We conducted a meta-analysis using EPT-specific results from the Collaborative Group on Hormonal Factors in Breast Cancer (CGHFBC) pooled analysis and studies published since that report to obtain an overview of EPT use and breast cancer risk. We also assessed risk by histologic subtype of breast cancer, by schedule of the progestin component of EPT, and by recency of use. We estimate that overall, EPT results in a 7.6% increase in breast cancer risk per year of use. The risk was statistically significantly lower in US studies than in European studies – 5.2 *vs* 7.9%. There was a significantly higher risk for continuous-combined than for sequential EPT use in Scandinavian studies where much higher total doses of progestin were used in continuous-combined than in sequential EPT. We observed no overall difference in risk for lobular *vs* ductal carcinoma but did observe a slightly higher risk for current *vs* past EPT use.

The Collaborative Group on Hormonal Factors in Breast Cancer (CGHFBC) (1997) pooled data from 51 epidemiologic studies to obtain an overall estimate of breast cancer risk associated with menopausal hormone therapy (HT) use. The risk estimate for oestrogen therapy (ET) use was based on large numbers of cases and controls, but the oestrogen–progestin therapy (EPT) result was not. Since then, a number of statistically powerful studies have evaluated EPT in relation to breast cancer risk. Some of these further evaluated differences in risk by schedule of progestin administration, that is, sequential *vs* continuous-combined use (Magnusson *et al*, 1999; Ross *et al*, 2000; Schairer *et al*, 2000; Newcomb *et al*, 2002; Porch *et al*, 2002; Weiss *et al*, 2002; Million Women Study, 2003; Olsson *et al*, 2003; Stahlberg *et al*, 2004), and in relation to histologic subtype of breast cancer (Schairer *et al*, 2000; Daling *et al*, 2002; Newcomb *et al*, 2002; Newcomer *et al*, 2003; Ursin *et al*, 2002; Weiss *et al*, 2002).

We conducted a meta-analysis of the results reported by the CGHFBC and studies published since that overview through March 2004 to provide a more precise estimate of the risk from EPT and how it is affected by schedule of progestin administration and histologic subtype.

## MATERIALS AND METHODS

We used the Medline database to compile a list of studies subsequent to the CGHFBC report investigating the relationship between EPT and incident breast cancer risk using the Medical Subject Headings (MeSH): postmenopausal, oestrogen progestin therapy (or combined therapy), and breast cancer. For this analysis we did not include studies that presented results only for overall HT, nor did we include studies that only evaluated ET use, nor studies only evaluating breast cancer mortality. A total of 22 studies were identified for possible inclusion (Persson *et al*, 1996, 1997, 1999; Magnusson *et al*, 1999; Li *et al*, 2000, 2002, 2003; Moorman *et al*, 2000; Rockhill *et al*, 2000; Ross *et al*, 2000; Schairer *et al*, 2000; Chen *et al*, 2002; Daling *et al*, 2002; Kirsh and Kreiger, 2002; Newcomb *et al*, 2002; Newcomer *et al*, 2003; Porch *et al*, 2002; Ursin *et al*, 2002; Weiss *et al*, 2002; Chlebowski *et al*, 2003; Jernstrom *et al*, 2003; Million Women Study, 2003; Olsson *et al*, 2003; Stahlberg *et al*, 2004). As age at menopause is a critical factor in assessing HT use and breast cancer risk (Pike *et al*, 1998), we excluded those studies that did not adjust for age at menopause: this criterion excluded five studies (Persson *et al*, 1997; Moorman *et al*, 2000; Chen *et al*, 2002; Li *et al*, 2002, 2003). We also excluded three studies that did not have information on risk by duration of EPT use (Persson *et al*, 1996; Li *et al*, 2000; Newcomer *et al*, 2003), and the study of Olsson *et al* (2003) for reasons described in the Discussion section below. We further excluded two studies since the results were based on the same data incorporated into the CGHFBC report (Persson *et al*, 1999; Rockhill *et al*, 2000), and the study of Jernstrom *et al* (2003), because it only provided results for continuous-combined EPT use, and the reason for this was the greater observed effect with such use than with sequential use. Therefore, the results from 10 recent studies and from the CGHFBC pooled analysis were used to obtain an overall assessment of EPT and breast cancer risk (CGHFBC, 1997; Magnusson *et al*, 1999; Ross *et al*, 2000; Schairer *et al*, 2000; Kirsh and Kreiger, 2002; Newcomb *et al*, 2002; Porch *et al*, 2002; Weiss *et al*, 2002; Chlebowski *et al*, 2003; Stahlberg *et al*, 2004).

In a second analysis, breast cancer risk by histologic subtype (lobular *vs* ductal) was evaluated in relation to EPT use. Four of the 10 studies had information on histology (Schairer *et al*, 2000; Daling *et al*, 2002; Newcomb *et al*, 2002; Ursin *et al*, 2002); Ursin *et al* (2002) used data from the study of Ross *et al* (2000) and Daling *et al* (2002) from the study of Weiss *et al* (2002). No information was available by histologic subtype from the CGHFBC report.

In a third analysis, we assessed breast cancer risk by progestin schedule (sequential, i.e. oestrogen given alone during the first part of a monthly cycle followed by oestrogen combined with a progestin for the remainder of the cycle with possibly a short hormone-free interval, *vs* continuous-combined, i.e. oestrogen and progestin always administered together during a cycle). No information was available by progestin schedule from the CGHFBC report on EPT use and breast cancer risk. Seven of the 10 studies had data subdivided by progestin schedule of EPT use (Magnusson *et al*, 1999; Ross *et al*, 2000; Newcomb *et al*, 2002; Porch *et al*, 2002; Weiss *et al*, 2002; Million Women Study, 2003; Stahlberg *et al*, 2004). However, two of these seven studies did not assess duration of use in relation to schedule and were omitted from this analysis (Newcomb *et al*, 2002; Porch *et al*, 2002).

As so few studies provided information on past HT use, we were limited in our ability to assess the difference in risk by recency of use. Of the 11 studies included in the overall analysis, four studies presented results comparing risk for past *vs* current HT use (Magnusson *et al*, 1999; Newcomb *et al*, 2002; Weiss *et al*, 2002; Million Women Study, 2003). Of these, three studies assessed risk by duration of past and current HT use and were included in the analysis (Magnusson *et al*, 1999; Weiss *et al*, 2002; Million Women Study, 2003), but only the study by Weiss *et al* (2002) reported risk for EPT use exclusively; the other two studies (Magnusson *et al*, 1999; Million Women Study, 2003) reported risk by past combined EPT and ET use.

Log odds ratios (LORs) per year of use (LOR_1_) and 95% confidence intervals (CI) were calculated for each study using the meta-analytic methods described by Greenland (Rothman and Greenland, 1998). The model fitted is log-linear in duration of EPT. (The hazard ratios calculated for the prospective and randomised trial studies closely approximate ORs and we refer to both as ORs in this paper.) For all analyses, the most fully adjusted multivariate odds ratios were used. The fixed-effects and random effects summary LOR_1_s were calculated by standard methods (DerSimonian and Laird, 1986; Fleiss, 1986). For all tables, we only present the fixed-effects LOR_1_s and provide two-sided *P*-values for heterogeneity as *P*_het_. All analyses including funnel plots (Begg and Mazumdar, 1994; Egger *et al*, 1997) were conducted using the *meta* and *metabias* commands in STATA (Stata Corporation, College Station, TX, USA).

The Women's Health Initiative (WHI) trial (Chlebowski *et al*, 2003) found, based on an intent-to-treat analysis, an average odds ratio (AOR) of invasive breast cancer of 1.24 for EPT use after an average of 5.6 years of follow-up. To convert this 1.24 figure into an OR_1_, we proceeded as follows: writing the *instantaneous* OR at the end of *d* years of use as OR_*d*_, then the AOR up to the end of year *t*, AOR_*t*_, is the integral of the OR_*d*_'s with *d* taking all values from 0 through *t* divided by the cumulative standardised risk in women not exposed to EPT, that is, *t*. This can be shown to result in the following equation: 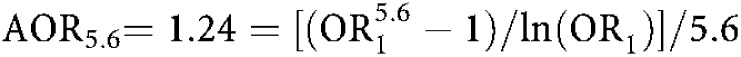 Solving this equation gives OR_1_=1.080.

For cohort studies, the true duration of EPT use is underestimated in current hormone users. This is because EPT use is assessed at baseline but continues for an unknown proportion of individuals for at least some further period until censoring time. Therefore, an additional duration of use should be added for current hormone users in the cohort studies considered (CGHFBC, 1997; Schairer *et al*, 2000; Porch *et al*, 2002; Million Women Study, 2003; Stahlberg *et al*, 2004). For example, in the cohort study of Porch *et al* (2002), they reported OR's of 1.11 and 1.76 for <5 and ⩾5 years of EPT use. We considered these categories as referring to 2.5 and 7.5 years of EPT use. Using these duration figures, we estimated OR_1_ as 1.079. But the ORs of 1.11 and 1.76 do not relate to 2.5 and 7.5 years of use, but to this amount of use *plus* the mean duration of use after recruitment to the study until the end of follow-up. The mean length of follow-up in this study was 5.9 years and assuming that current users of EPT remained users during follow-up, this changes the values to be used in estimating OR_1_ from 2.5 and 7.5 years to 5.45 (2.5 plus the midpoint of the average follow-up, i.e. 5.9/2 or the average exposure during follow-up) and 10.45 (7.5 + 5.9/2) years, respectively. This changes our estimate of OR_1_ from 1.079 to 1.052, a 34% decline in our estimate of excess risk. This is, of course, a slight exaggeration of the change since some current users at baseline will stop use during follow-up. For all cohort studies included in the analysis, we calculated risk per year of use based on this conservative method. We applied this method to all prospective studies reporting risk for current EPT use except for the study by Schairer *et al* (2000), in which this adjustment had already been applied.

Risk estimates reported in the study by Magnusson *et al* (1999) were converted to risks per year of use since the OR_1_s reported in the study excluded never users of EPT. This was done in order for these estimates to be comparable to the relative risks reported in the other studies.

## RESULTS

The studies included in at least one of the three analyses conducted to evaluate EPT and breast cancer risk are given in Appendix A. A summary table of the general characteristics and overall findings for each study are presented in Appendix B. As is apparent in the summary table, the effect of EPT use by duration of use in these various studies was evaluated in a wide variety of ways with categorical cutpoints, as well as per year of use.

### EPT and breast cancer risk

**Figure 1:**
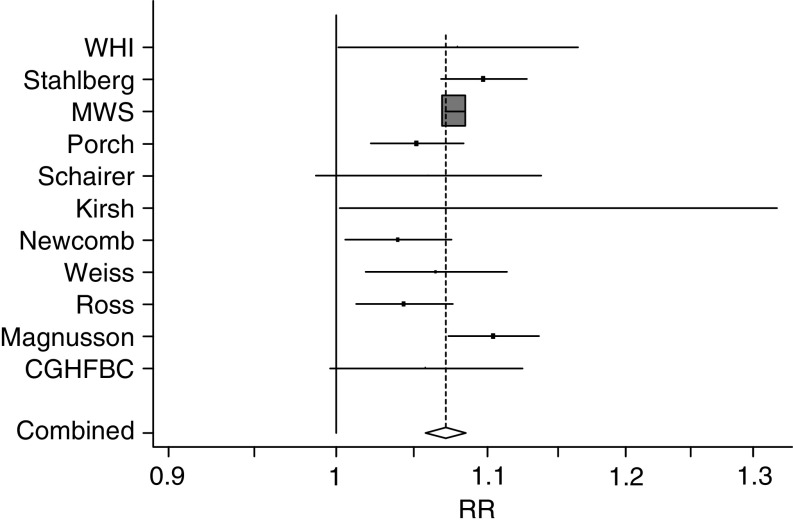
Studies included in overall analysis of EPT and risk of breast cancer: odds ratios with 95% CIs per year of use.

The overall summary of the studies included in this meta-analysis (all histologic subtypes combined) showed a weighted average OR_1_ of 1.076. (95% confidence interval (CI)=1.070, 1.082) for EPT use, with some evidence of heterogeneity, *P*_het_=0.074 (Table 1, Figure 1). A funnel plot showed no evidence of publication bias. The OR_1_ for the US studies was 1.052 (95% CI=1.036, 1.068); for the European studies was 1.079 (95% CI=1.073, 1.085); and for the Scandinavian studies was 1.089 (95% CI=1.065, 1.114): the difference between the US studies and the European studies was highly statistically significant (*P*=0.002) (Table 1).

### EPT by sequential *vs* continuous-combined schedules and breast cancer risk

Sequential EPT use was associated with a lower OR_1_ than continuous-combined EPT use (Table 2). The best estimate of the overall difference between the OR_1_s was −0.015 (95% CI=−0.030, 0.000), *P*_diff_=0.054. The most obvious difference between the continuous-combined and sequential schedules was seen in the two Scandinavian studies (Magnusson *et al*, 1999; Stahlberg *et al*, 2004) in which the difference in OR_1_s was −0.065 (95% CI=−0.115, −0.015), *P*_diff_=0.010. In the remaining studies the average OR_1_ difference was −0.010 (95% CI=−0.026, 0.006), *P*_diff_=0.23; this figure essentially reflects the Million Women Study (2003) in which the difference was −0.013 whereas the remaining two studies (Ross *et al*, 2000; Weiss *et al*, 2002) had differences of −0.056 and 0.050.

### EPT and lobular *vs* ductal breast cancer risk

Two of the four studies evaluating the difference in risk between lobular and ductal breast carcinoma found no difference by histology while the other two studies observed a slightly increased risk for lobular carcinoma (Table 3). The overall difference in risk (at one year) by histologic subtype was 0.019 (95% CI=−0.033, 0.071), *P*_diff_=0.47.

### Current/recent use *vs* total lifetime use and breast cancer risk

Only three studies (Magnusson *et al*, 1999; Weiss *et al*, 2002; Million Women Study, 2003) reported results comparing risk for past *vs* current HT use. Pooled estimates for these three studies showed that the difference in OR_1_s was −0.067 (95% CI=−0.081, −0.053), *P*_diff_<0.001 (Table 4). In the study by Weiss *et al* (2002), the only study to report risk separately for past and current EPT use, the difference was −0.100 (−0.166, −0.034), *P*_diff_=0.003.

As another way to assess potential difference in risk by recency of use, we calculated pooled estimates for those studies reporting relative risks among current/and or recent EPT use (CGHFBC, 1997; Schairer *et al*, 2000; Porch *et al*, 2002; Chlebowski *et al*, 2003; Million Women Study, 2003; Stahlberg *et al*, 2004) and compared them to pooled estimates for the studies reporting risk for lifetime EPT use (Magnusson *et al*, 1999; Ross *et al*, 2000; Kirsh and Kreiger, 2002; Newcomb *et al*, 2002; Weiss *et al*, 2002). The pooled estimate (data not shown) for the studies assessing current/recent use was slightly higher (OR_1_=1.077, 95% CI=1.071, 1.083) than the pooled estimate for studies reporting lifetime EPT use (OR_1_=1.053, 95% CI=1.034, 1.072), and this difference was statistically significant, *P*_diff_=0.019. This difference remained significant even after excluding the Scandinavian studies (Magnusson *et al*, 1999; Stahlberg *et al*, 2004): the weighted average OR_1_ for current/recent use was 1.076 (95% CI=1.070, 1.082) and 1.049 (95% CI=1.028, 1.070) for lifetime use, *P*_diff_=0.017.

## DISCUSSION

The literature evaluating EPT and breast cancer risk is generally very consistent; all studies reported an increased risk of breast cancer with increasing duration of EPT use. The overall evidence showed a statistically significant increased risk of 7.6% per year of use. The risk was statistically significantly lower in US studies than in European studies – 5.2 *vs* 7.9%.

The US figure should probably be increased slightly since the results we used for the WHI trial (Chlebowski *et al*, 2003) are almost certainly an underestimate of the true effect in that trial. We used the result obtained from their intent-to-treat analysis. The results from their drug-as-taken analysis was double that obtained from the intent-to-treat analysis. The WHI trial result has, however, only a small effect on the overall risk for US studies as it is associated with a wide CI. The results of the Scandinavian study of Stahlberg *et al* (2004) are likewise likely to be an underestimate, although to a smaller extent than with the WHI, as the authors only adjusted for age at menopause as <55 and ⩾55 years.

The overall within-study difference between sequential *vs* continuous-combined EPT was −0.015 (95% CI=−0.030, 0.000), *P*_diff_=0.054. This difference was due to the two Scandinavian studies (Magnusson *et al*, 1999; Stahlberg *et al*, 2004) where the risk was −0.065 (95% CI=−0.115, −0.015), *P*_diff_=0.010. This difference was also supported by the results of the Scandinavian study reported by Jernstrom *et al* (2003), which we excluded earlier since the authors only presented results for continuous-combined EPT use; the authors reported their results in this manner because of the greater observed effect for continuous-combined than for sequential EPT use.

In the US, the most common form of sequential EPT provides 5–10 mg of medroxyprogesterone acetate (MPA) per day for 10 days per 28-day cycle, whereas subjects assigned to receive continuous-combined EPT are typically given 2.5 mg of MPA every day. The total doses for sequential and continuous-combined are therefore very close at approximately 75 and 70 mg respectively per cycle. In contrast, in Scandinavia, the total dose of the progestin is much higher with continuous-combined than with sequential EPT, at least for two commonly prescribed regimens using norethisterone acetate (NETA). In these regimens, the same daily dose of NETA, 1 mg, is used with both the sequential and the continuous-combined EPT, so that the total NETA dose per cycle is roughly 10 and 28 mg respectively. The situation in the UK is more like that in the US, with the continuous-combined regimens using lower daily progestin doses than that used in the sequential regimens, so that total progestin doses are not that different. This would be in agreement with the results found by the Million Women Study (2003).

Some of the difference in the risks found between the US studies and the European studies are due to the higher risks found with sequential regimens in Scandinavia and likely due to higher total doses of progestin (as described above). The remainder may be due to the much greater use of NETA and norgestrel in Europe. Based on its effects in the endometrium (Dickey and Stone, 1976; Back *et al*, 1981; Stanczyk, 2002), the progestin dose of NETA as commonly prescribed is possibly 1.5–2.0 times the effective dose of progestin used in the US. There is also the possibility that the different types and doses of oestrogen used have different effects. Finally, some of the differences in risk may be due to a greater relative effect of HT use on breast cancer risk among leaner women. The women in the US studies in this meta-analysis are in general heavier than the women in the European studies, consistent with overall population demographics. In this meta-analysis we did not assess differences in risk by weight as only two studies evaluated risk with duration of EPT use (Schairer *et al*, 2000; Ursin *et al*, 2002) and the study of Ursin *et al* with much larger numbers found no differential effect of BMI; the others only gave results with duration of HT use (Magnusson *et al*, 1999), or with ever HT or EPT use (CGHFBC, 1997; Newcomb *et al*, 2002; Ursin *et al*, 2002; Weiss *et al*, 2002; Million Women Study, 2003).

Summary risk estimates by histology were higher for lobular than for ductal carcinoma; the OR_1_ difference was 0.019, but this was not statistically significant. Further data are needed on this issue.

Schairer *et al* (2000) reported much higher estimates than the other studies for both lobular (OR_1_=1.17) and ductal carcinoma (OR_1_=1.17). These risks compare to their overall result (Table 1) of an OR_1_ of 1.076. The explanation is that the authors only provided results by histology among lean women, and in their study the effect of EPT on risk was much greater in lean women.

Only the study by Weiss *et al* (2002) compared risk for current *vs* past EPT use. Their results suggest that risk for current EPT use is higher. The Million Women Study (2003) compared current HT use and past EPT use, and the study by Magnusson *et al* (1999) compared current and past HT use. The results from these two studies are difficult to interpret since HT use in past users includes proportionately more ET use. We found that recent EPT use was associated with a higher risk than lifetime EPT use, but this analysis was based on only a small number of studies.

The observed lower risk with past use may be due, at least in part, to the fact that that duration of hormone use is not measured the same in current as in past hormone users. The actual duration of use within a duration category will tend to be longer in current than in past users (Ettinger *et al*, 2003), and, in cohort studies, duration of use is underestimated in current users since exposure is only assessed at baseline. Nondifferential misclassification of duration of use is also likely to be higher with past use, leading to a greater underestimate of the risk associated with past use. Four of the five studies reporting risk among current/recent users addressed the possibility that a screening bias could be a possible explanation for the observed lower risk among past users (Schairer *et al*, 2000; Porch *et al*, 2002; Rossouw *et al*, 2002; Chlebowski *et al*, 2003; Million Women Study, 2003). None found any evidence of this.

## Figures and Tables

**Table 1:** Odds ratios per year of use (OR_1_s) of oestrogen–progestin therapy and breast cancer risk

**Study**	**Case users**	**OR_1_ (95% CI)**	
*Randomised trial*			
WHI^1^ (2003)^a^	199	1.080 (1.004, 1.167)	

*Prospective studies*			
Stahlberg^2^ (2004)^a^^,^^b^	95	1.097 (1.068, 1.127)	
MWS^3^ (2003)^a^^,^^c^	1891	1.077 (1.071, 1.084)	
Porch^4^ (2002)^a^^,^^b^^,^^c^	164	1.052 (1.022, 1.084)	
Schairer^5^ (2000)^a^^,^^d^	75	1.060 (0.998, 1.150)	

*Case–control studies*			
Kirsh^6^ (2002)^b^^,^^c^	43	1.15 (1.01, 1.33)	
Newcomb^7^ (2002)^c^	215	1.04 (1.01, 1.08)	
Weiss^8^ (2002)	195	1.065 (1.019, 1.114)	
Ross^9^ (2000)^b^	425	1.044 (1.014, 1.077)	
Magnusson^10^ (1999)^b^	399	1.104 (1.073, 1.136)	

*Pooled studies*			
CGHFBC^11^ (1997)^a^^,^^e^	194	1.058 (0.996, 1.124)	

*Summary*		*Pooled estimate*	
All studies		1.076 (1.070, 1.082)	*P*_het_=0.074
US studies^1, 4−9,11^		1.052 (1.036, 1.068)	*P*_het_=0.87
European studies^2,3,10^		1.079 (1.073, 1.085)	*P*_het_=0.12
Scandinavian studies^2,10^		1.089 (1.065, 1.114)	*P*_het_=0.32

Abbreviations: CI=confidence interval; OR=odds ratio; WHI=Women's Health Initiative; MWS=Million Women Study; CGHFBC=Collaborative Group on Hormonal Risk Factors in Breast Cancer.

a Risk is based on current and/or recent use rather than total use.

b Results included (or did not specifically exclude) *in situ* breast cancer cases.

c Results included (or did not specifically exclude) women with unknown age at menopause due to simple hysterectomy.

d Calculated number of cases for women with known age at menopause: 80% of the total number of cases (*n*=93).

e Includes mostly US studies.

**Table 2:** Odds ratios per year of use (OR_1_s) of oestrogen–progestin therapy and breast cancer risk by progestin schedule

	**Sequential**		**Continuous**		
**Study**	**Case users**	**OR_1_ (95% CI)**	**Case users**	**OR_1_ (95% CI)**	**Difference**
*Prospective studies*					
Stahlberg *et al*^2^ (2004)^a^^,^^b^	29	1.063 (1.024, 1.103)	20	1.137 (1.093, 1.182)	−0.074 (−0.134, −0.014)
MWS^3^ (2003)^a^^,^^c^	1181	1.093 (1.083, 1.103)	631	1.106 (1.093, 1.120)	−0.013 (−0.030, 0.004)

*Case–control studies*					
Weiss^8^ (2002)	78	1.031 (0.966, 1.100)	166	1.087 (1.020, 1.159)	−0.056 (−0.153, 0.041)
Ross^9^ (2000)^b^	320	1.067 (1.025, 1.109)	105	1.017 (0.975, 1.062)	0.050 (−0.010, 0.110)
Magnusson^10^ (1999)^b^	102	1.088 (1.022, 1.158)	135	1.132 (1.072, 1.197)	−0.044 (−0.136, 0.048)

*Summary*		*Pooled estimate*		*Pooled estimate*	*Pooled estimate*
All studies		1.089 (1.080, 1.098)		1.103 (1.092, 1.115)	−0.015 (−0.030, 0.000)
		*P*_het_=0.20		*P*_het_=0.002	*P*_diff_=0.054

US studies^8,9^		1.057 (1.022, 1.093)		1.038 (1.002, 1.076)	+0.020 (−0.031, 0.071)
		*P*_het_=0.38		*P*_het_=0.090	*P*_diff_=0.44
European studies^2,3,10^		1.091 (1.082, 1.101)		1.110 (1.098, 1.122)	−0.018 (−0.034, −0.002)
		*P*_het_=0.36		*P*_het_=0.33	*P*_diff_=0.024
Scandinavian studies^2,10^		1.070 (1.036, 1.104)		1.135 (1.100, 1.172)	−0.065 (−0.115, −0.015)
		*P*_het_=0.53		*P*_het_=0.92	*P*_diff_=0.010

Abbreviations: CI=confidence interval; OR=odds ratio; WHI=Women's Health Initiative; MWS=Million Women Study; CGHFBC=Collaborative Group on Hormonal Risk Factors in Breast Cancer.

a Risk was based on current and/or recent use rather than total use.

b Risk included (or did not specifically exclude) *in situ* breast cancer cases.

c Results included (or did not specifically exclude) women with unknown age at menopause due to simple hysterectomy.

**Table 3:** Odds ratios per year of use (OR_1_s) of oestrogen–progestin therapy and breast cancer risk by histologic subtype

	**Lobular**		**Ductal**		
**Study**	**Case users**	**OR_1_ (95%CI)**	**Case users**	**OR_1_ (95%CI)**	**Difference**
*Prospective studies*					
Schairer^5^ (2000) a ^,^ b ^,^ c	33	1.17 (1.02, 1.41)	26	1.17 (1.02, 1.41)	0.000 (−0.276, 0.276)

*Case–control studies*					
Daling^13^ (2002)	44	1.096 (1.007, 1.193)	209	1.039 (0.99, 1.089)	0.057 (−0.048, 0.162)
Newcomb^7^ (2002) a	32	1.04 (0.97, 1.11)	208	1.04 (1.00, 1.08)	0.000 (−0.109, 0.109)
Ursin^14^ (2002)	46	1.060 (0.996, 1.128)	291	1.049 (1.016, 1.084)	0.011 (−0.063, 0.085)

*Summary*		*Pooled estimate*		*Pooled estimate*	*Pooled estimate*
		1.067 (1.026, 1.110)		1.046 (1.023, 1.069)	0.019 (−0.033, 0.071)
		*P*_het_=0.53		*P*_het_=0.56	*P*_diff_=0.47

Abbreviations: CI=confidence interval; OR=odds ratio; WHI=Women's Health Initiative; MWS=Million Women Study; CGHFBC=Collaborative Group on Hormonal Risk Factors in Breast Cancer.

a Results included (or did not specifically exclude) women with unknown age at menopause due to simple hysterectomy.

b Risk was based on current and/or recent use rather than total use.

c Risk among lean women, lobular/ductal *vs* ductal only, results included (or did not specifically exclude) *in situ* breast cancer.

**Table 4:** Odds ratios per year of use (OR_1_s) of oestrogen–progestin therapy and breast cancer risk by recency of use

	**Past**		**Current**		
**Study**	**Case users**	**OR_1_ (95% CI)**	**Case users**	**OR_1_ (95% CI)**	**Difference**
*Prospective studies*					
MWS^3^ (2003)^a^	1005	1.010 (0.998, 1.023)	1891	1.077 (1.071, 1.084)	−0.067 (−0.081, −0.053)

*Case–control studies*					
Weiss^8^ (2002)	187	0.947 (0.892, 1.005)	502	1.047 (1.013, 1.083)	−0.100 (−0.166, −0.034)

Magnusson^10^ (1999)^a^^,^^b^	200	1.065 (1.014, 1.118)	444	1.144 (1.109, 1.181)	−0.079 (−0.142, −0.016)

*Summary*		*Pooled estimate*		*Pooled estimate*	*Pooled estimate*
All studies		1.011 (0.999, 1.023)		1.079 (1.073, 1.085)	−0.069 (−0.082, −0.055)
		*P*_het_=0.011		*P*_het_<0.001	*P*_diff_<0.0001

Abbreviations: CI=confidence interval; OR=odds ratio; MWS=Million Women Study.

a Risk for ET or EPT use.

b Risk included (or did not specifically exclude) *in situ* breast cancer cases.

**Table tbla1:** 

	**Analysis 1**	**Analysis 2**		**Analysis 3**		**Analysis 4**	
**Study**	**Overall**	**Lobular**	**Ductal**	**Sequential**	**Continuous-combined**	**Past**	**Current**
*Randomised Trials*							
WHI^1^ (2003)	X				c		

*Prospective studies*							
Stahlberg^2^ (2004) a	X			X	X		
MWS^3^ (2003)	X			X	X	X^e^	X
Porch^4^ (2002)	X			d	d		
Schairer^5^ (2000)	X	X	X	c			

*Case–control studies*							
Kirsh^6^ (2002)	X						
Newcomb^7^ (2002)	X	X	X	d	d	d	d
Weiss^8^ (2002)	X	X	X	X	X	X	X
Daling ^13^ (2002) b							
Ross^9^ (2000)	X	X	X	X	X		
Ursin^14^ (2002) b							
Magnusson^10^ (1999)	X			X	X	X^e^	X^e^

*Pooled studies*							
CGHFBC^11^ (1997)	X						

Abbreviations: CI=confidence interval; OR=odds ratio; WHI=Women's Health Initiative; MWS=Million Women Study; CGHFBC=Collaborative Group on Hormonal Risk Factors in Breast Cancer.

a Overall risk calculated from sequential and continuous-combined use.

b Results by histologic subtype, not overall breast cancer risk (overall risk for Daling in Weiss and for Ursin in Ross).

c Only results for one type of progestin schedule.

d No results given for duration of EPT use.

e Risk for ET or EPT use.

**Table tbla2:** 

	**Randomised trials**		**Cases**	**Population**	**Mean follow-up (years)**	**Adjusted variables**	**Results**
1	WHI^1^ (2003)	Healthy postmenopausal women in the Women's Health Initiative Trial	199 treatment 150 placebo	8506 treatment 8102 placebo	5.6	Age, dietary modification, randomisation group	1.24 (1.01, 1.54) cumulative risk^a^


	**Prospective studies**	**Study population^b^**	**Cases**	**Person-years**	**Mean follow-up (years)**	**Adjusted variables**	**Results**
2	Stahlberg^2^ (2004)	Healthy postmenopausal women age 45+ in the Danish Nurse Cohort	139^c^	7572^d^	6.34	Age, hx of BBD, age at meno (<55 *vs* ⩾55)	Sequential :<5 yr: 1.58 (0.79, 3.17) 5–9 yr: 2.47 (1.23, 4.95) 10+ yr: 2.18 (1.09, 4.33)^a^
			130^c^	6889^d^			Continuous-combined: <5 yr: 1.96 (0.72, 5.36) 5-9 yr: 4.96 (2.16, 11.39) 10+ yr: 6.78 (3.41, 13.48)^a^

3	MWS^3^ (2003)	Healthy women^e^ in the UK age 50–64 as part of the National Health Service Breast Screening Programme	4785	532 353^d^	2.6	Age, time since meno, parity, AFFTP, family hx of breast cancer, BMI, region, deprivation index	<1 yr: 1.45 (1.19, 1.78) 1–4 yr: 1.74 (1.60, 1.89) 5–9 yr: 2.17 (2.03, 2.33) ⩾10 yr: 2.31 (2.08, 2.56)^a^
			4075	478 399^d^			Sequential: <5 yr: 1.77 (1.59, 1.97) ⩾5 yr: 2.12 (1.95, 2.30)^a^
			3525	441 751^d^			Continuous-combined: <5 yr: 1.57 (1.37, 1.79) ⩾5 yr: 2.40 (2.15, 2.67)^a^
			1005	144 697^d^			Past HT: <1 yr: 0.94 (0.84, 1.05) 1–4 yr: 1.01 (0.92, 1.12) 5–9 yr: 1.14 (1.00, 1.30) ⩾10 yr: 1.05 (0.84, 1.30) Current EPT: s*ee above results for overall analysis*

4	Porch^4^ (2002)	Healthy female postmenopausal^e^ professionals age 45+ in the Women's Health Study	310^c^	71 438	5.9	Age, age at meno, meno type, age at menarche, nulliparity, age at first preg, abortions/miscarriages, AFFTP, OC, hx of BBD, use of mammo screening, family hx breast cancer, race, BMI, smoking, alcohol, exercise	<5 yr: 1.11 (0.81, 1.52) 5+ yr: 1.76 (1.29, 2.39) * P*_trend_=0.0004^a^

5	Schairer^5^ (2000)	Healthy postmenopausal^e^ women in the Breast Cancer Detection Demonstration Project	∼75 (known age meno)	∼15 727(known age meno)	10.2 (over three phases of follow-up)	Age, edu, BMI, age at meno, mammo screening	1.06 (1.00, 1.15) per year of use among known age meno^a^
			33	18 948			Lobular/ductal: 1.17 (1.02, 1.41) per year of use among all women^a^^,^^f^
			26	18 948			ductal: 1.17 (1.02, 1.41) per year of use among all women^a^^,^^f^
			48	18 948			Sequential: (*P*<15 days/mo) ⩽4 yr: 1.1 (0.8, 1.7) >4 yr: 1.5 (1.0, 2.4) among all women^a^


	**Case–control studies**	Study population^*^	**Cases**	**Controls**		**Adjusted variables**	**Results**
6	Kirsh^6^ (2002)	Healthy postmenopausal^e^ women in Ontario, Canada	404^c^	403		Age, age at meno, type of meno, hx of BBD	1.15 (1.01, 1.33) per year of use
7	Newcomb^7^ (2002)	Healthy postmenopausal^e^ women from Massachusetts, New Hampshire, Wisconsin	4142	4418		Age at meno, type of meno, AFFTP, BMI, family hx breast cancer, edu, mammo screening hx, recent alcohol, hx of BBD, age at menarche, recent physical activity	1.04 (1.01, 1.08) per year of use
			351	4132			Lobular: 1.04 (0.97, 1.11) per year of use
			2654	4132			Ductal: 1.04 (1.00, 1.08) per year of use
8	Weiss^8^ (2002)	Healthy black and white postmenopausal women in Atlanta, Detroit, Los Angeles, Philadelphia, Seattle in the Contraceptive and Reproductive Experiences (CARE) Study	849	835		Age, race, study center, type of meno, age at meno	>0 to <6 mo: 0.59 (0.40, 0.87) 6 mo to <2 yr: 0.82 (0.57, 1.18) 2 to <5 yr: 1.33 (0.91, 1.95) 5+ yr: 1.49 (1.05, 2.12)
			672	661			Sequential: (*P*<25 days/mo) >0 to <6 mo: 0.70 (0.38, 1.30) 6 mo to <2 yr: 0.72 (0.42, 1.24) 2 to <5 yr: 1.44 (0.79, 2.61) 5+ yr: 1.18 (0.70, 1.98)
			730	696			Continuous-combined: (*P*⩾25 days/mo) >0 to <6 mo: 0.58 (0.36, 0.94) 6 mo to <2 yr: 1.11 (0.71, 1.75) 2 to <5 yr: 1.38 (0.86, 2.22) 5+ yr: 1.77 (1.04, 3.01)
			859	899			Past EPT :>0 to <6 mo: 0.68 (0.47, 0.99) 6 mo to <2 yr: 0.76 (0.52, 1.13) 2 to <5 yr: 1.24 (0.79, 1.93) 5+ yr: 0.54 (0.33, 0.88)
			1174	1042			Current EPT: >0 to <6 mo: 0.53 (0.26, 1.09) 6 mo to <2 yr: 1.11 (0.75, 1.65) 2 to <5 yr: 1.28 (0.95, 1.73) 5+ yr: 1.37 (1.06, 1.77)

	Daling^13^ (2002)		108	835		Age, race, study site, type of meno, known age at meno	Lobular: ⩽6 mo: 0.7 (0.3, 1.5) 6 mo to 5 yr: 1.3 (0.7, 2.3) 5 yr+: 1.9 (1.0, 3.7)
			635	835			Ductal: ⩽6 mo: 0.6 (0.4, 0.9) 6mo to 5 yr: 1.0 (0.7, 1.3) 5 yr+: 1.3 (0.9, 1.9)
9	Ross^9^ (2000)	Healthy postmenopausal women in Los Angeles	1298^c^	1108		Type of meno, age at meno, age at menarche, family hx of breast cancer, history BBD, nulliparity, AFFTP, OCs, BMI, alcohol	1.24 (1.07, 1.45) per 5 yr of use* P*_2-sided_=0.005
			1193^c^	1012			Sequential: (*P*<entire cycle) 1.38 (1.13, 1.68)* P*_2-sided_=0.0015
			978^c^	880			Continuous-combined: (*P*=entire cycle) 1.09 (0.88, 1.35)* P*_2-sided_=0.44
	Ursin^14^ (2002)		164	1637		Type and age at meno, age at menarche, family hx breast cancer, hx of BBD, nulliparity, AFFTP, OCs, weight, alcohol	Lobular:1.34 (0.98, 1.83) per 5 yr of use* P*_trend_=0.06
			1307	1637			Ductal: 1.27 (1.08, 1.50) per 5 yr of use* P*_trend_=0.004

10	Magnusson^10^ (1997)	Healthy postmenopausal women in Sweden	2137^c^	2481		Age, parity, AFFTP, age at meno, type of meno, BMI, height	1–24 mo: 1.25 (0.96, 1.63) 25–60 mo: 1.40 (1.01, 1.94) 60–120 mo: 2.43 (1.72, 3.44) >120 mo: 2.95 (1.84, 4.72)
			1841^c^	2272			Sequential: (*P*<16 days/mo) 1–24 mo: 1.58 (1.01, 2.46) 25–60 mo: 1.34 (0.71, 2.54) 60–120 mo: 1.89 (0.88, 4.09) >120 mo: 2.45 (0.82, 7.30)
			1874^c^	2322			Continuous-combined: (*P*⩾19 days/mo) 1–24 mo: 0.93 (0.63, 1.36) 25–60 mo: 1.26 (0.76, 2.09) 60–120 mo: 2.89 (1.66, 5.00) >120 mo: 5.36 (1.47, 19.56)
			1828^c^	2302			Past HT: (1–10 yr ago): 1–60 mo: 1.09 (0.76, 1.55) >60 mo: 1.22 (0.72, 2.08) (> 10 yr ago): 1–60 mo: 1.24 (0.90, 1.70) >60 mo: 2.57 (1.28, 5.15)
			2182^c^	2475			Current HT (<1 yr ago): 1–60 mo: 1.52 (1.21, 1.92) >60 mo: 2.68 (2.09, 3.42)


	**Pooled analysis**	Study population*	**Cases**	**Controls**		**Adjusted variables**	**Results**
11	CGHFBC^11^ (1997)	Women from 22 published and two unpublished studies with information on type of preparation (EPT, ET, PT)	12 611	23 866		Study, age at dx or pseudo dx, time since meno, BMI, parity, AFFTP	<5 yr: 1.15 (s.e.=0.19) ⩾5 yr: 1.53 (s.e.=0.33)^a^

Abbreviations: WHI=Women's Health Initiative; MWS=Million Women Study; CGHFBC=Collaborative Group on Hormonal Risk Factors in Breast Cancer; OC=oral contraceptives; hx=history; BBD=benign breast disease; BMI=body mass index, preg=pregnancy; mammo=mammography; edu=education level; meno=menopause; AFFTP=age at first full-term pregnancy; dx=diagnosis; mo=month(s); yr=year(s); P=progestin; E=oestrogen; s.e.=standard error.

a Risk was based on current and/or recent use rather than total use.

b Number of cases and number of starting population/person-years/controls for the results presented in final column.

c Results included (or did not specifically exclude) *in situ* breast cancer cases.

d Authors provided population rather than person-years.

e Results included (or did not specifically exclude) women with unknown age at menopause due to simple hysterectomy.

f Risk among lean women.

## Appendix A

Studies evaluating oestrogen–progestin use and breast cancer risk. Studies included in each analysis are represented by an ‘ × ’ and studies excluded are marked by footnotes.

[Table tbla1]

## Appendix B

Studies evaluating oestrogen–progestin use and breast cancer risk: overall characteristics and main findings.

[Table tbla2]
